# Prenatal exposure to heavy metal mixtures and anthropometric birth outcomes: a cross-sectional study

**DOI:** 10.1186/s12940-022-00950-z

**Published:** 2022-12-29

**Authors:** Tal Michael, Elkana Kohn, Sharon Daniel, Ariela Hazan, Matitiahu Berkovitch, Anna Brik, Ori Hochwald, Liron Borenstein-Levin, Moshe Betser, Miki Moskovich, Ayelet Livne, Rimona Keidar, Efrat Rorman, Luda Groisman, Zeev Weiner, Adi Malkoff Rabin, Ido Solt, Amalia Levy

**Affiliations:** 1 Department of Epidemiology, Biostatistics, and Community Health Sciences, School of Public Health, Faculty of Health Sciences, Ben-Gurion University of the Negev Beer-Sheva, Beersheba, Israel; 2 Clinical Pharmacology and Toxicology Unit, Pediatric Division, Shamir (Assaf Harofeh) Medical Center, and Sackler School of Medicine, Tel-Aviv University, Tel-Aviv, Israel; 3 Clalit Health Services, Southern District, Beer-Sheva, Israel; 4 Neonatal Intensive Care Unit, Rambam Health Care Campus, and Bruce Rappaport Faculty of Medicine, Technion, Israel Institute of Technology, Haifa, Israel; 5 Delivery Rooms and Maternity Ward, Shamir (Assaf Harofeh) Medical Center, and Sackler School of Medicine, Tel-Aviv University, Tel-Aviv, Israel; 6 Neonatal Intensive Care Unit, Shamir (Assaf Harofeh) Medical Center, Sackler School of Medicine, Tel-Aviv University, Tel-Aviv, Israel; 7 National Public Health Laboratory, Ministry of Health, Tel-Aviv, Israel; 8 Department of Obstetrics and Gynecology, Rambam Health Care Campus and Bruce Rappaport Faculty of Medicine, Technion, Israel Institute of Technology, POB 9602, 31096 Haifa, Israel; 9 Environment and Health Epidemiology Research Center, School of Public Health, Faculty of Health Sciences, Ben-Gurion University of the Negev, Beer-Sheva, Israel

**Keywords:** Anthropometric Measures, Prenatal Exposure, Pregnancy, Metals, BKMR

## Abstract

**Background:**

Numerous studies have suggested significant associations between prenatal exposure to heavy metals and newborn anthropometric measures. However, little is known about the effect of various heavy metal mixtures at relatively low concentrations. Hence, this study aimed to investigate associations between prenatal exposures to a wide range of individual heavy metals and heavy metal mixtures with anthropometric measures of newborns.

**Methods:**

We recruited 975 mother–term infant pairs from two major hospitals in Israel. Associations between eight heavy metals (arsenic, cadmium, chromium, mercury, nickel, lead, selenium, and thallium) detected in maternal urine samples on the day of delivery with weight, length, and head circumference at birth were estimated using linear and Bayesian kernel machine regression (BKMR) models.

**Results:**

Most heavy metals examined in our study were observed in lower concentrations than in other studies, except for selenium. In the linear as well as the BKMR models, birth weight and length were negatively associated with levels of chromium. Birth weight was found to be negatively associated with thallium and positively associated with nickel.

**Conclusion:**

By using a large sample size and advanced statistical models, we could examine the association between prenatal exposure to metals in relatively low concentrations and anthropometric measures of newborns. Chromium was suggested to be the most influential metal in the mixture, and its associations with birth weight and length were found negative. Head circumference was neither associated with any of the metals, yet the levels of metals detected in our sample were relatively low. The suggested associations should be further investigated and could shed light on complex biochemical processes involved in intrauterine fetal development.

**Supplementary Information:**

The online version contains supplementary material available at 10.1186/s12940-022-00950-z.

## Introduction

Heavy metals are naturally occurring elements with a high atomic weight and density at least five times greater than water. Some of these heavy metals are essential nutrients in the body, and a deficiency in one of them can result in diseases [1]. On the other hand, over-consumption and exposure to high levels of heavy metals have been associated with adverse health outcomes [2–4]. Over-exposure of both mother and fetus to heavy metals during pregnancy[5] has been associated with preterm birth and reduced birth size [6–8].

While the mechanisms underlying the effect of over-exposure to heavy metals on the development of newborns remain the subject of ongoing studies [9, 10], some heavy metals, including cadmium (Cd), mercury (Hg), lead (Pb), and selenium (Se), have been found to cross the placental barrier [11] and accumulate in the fetal blood circulation. The associations between prenatal exposure to these metals and adverse birth outcomes have been widely studied and raised possible associations with shorter birth length [12], low birth weight [13], and small head circumference [14]. Prenatal exposure to other metals, such as arsenic (As), thallium (Tl), nickel (Ni), and chromium (Cr), has been less extensively studied but was also found to be associated with various adverse birth outcomes [15–17]. Prenatal assessment of heavy metal exposures during pregnancy is challenging and is usually conducted by analysis of maternal blood, assuming exposure traces found in it are highly correlated with human cord blood levels, as previously shown by Kot et al. (2021) [18] and Li et al. (2018) [19]. The latter examined the efficiency of placental transfer of several metals and suggested that while Cr and As accumulated in the blood cord easily, the accumulation of others including Ni is less prominent. Maternal exposure can also be monitored by examination of metal traces in urine. Although very few studies have examined the correlation between metal traces found in maternal urine and cord blood [20], a relatively high correlation between metals found in urine and maternal blood was reporeted [21]. Thus, Ashrap et al. (2021) suggested measuring metals in either urine or maternal blood may be an equally good approach to evaluate associations with intrauterine exposures [22].

In recent years, many epidemiologic studies have examined the associations between heavy metals measured in maternal urine and various adverse health outcomes among newborns, including low birth weight [23], low birth size [6], and various congenital abnormalities [24]. While these findings alone may be associated with morbidity in early childhood [25] and adulthood [26], they may have resulted from a complicated sequence of intrauterine events [27] that could be associated with many other future complications, including behavioral changes in early childhood [28], obesity during late childhood [29] and various endocrine disruptions [30]. Hence, it is crucial to investigate any associations between prenatal exposure to various heavy metals and measurable and sensitive birth outcomes. Until now, most studies conducted in this field have focused on populations exposed to relatively high levels of heavy metals [31, 32], rather than levels similar to the average background of exposure, where no exceptional exposures occur.

In the current study, we examine the association between prenatal exposure to a mixture of heavy metals (as measured in maternal urine) and newborn anthropometric measures. We investigated the concentrations of eight heavy metals (As, Cd, Cr, Hg, Ni, Pb, Se, and Tl) in maternal urine samples and examined their association with anthropometric measures, both individually and by using a modeling approach that accounts for possible non-linear associations, as well as any interactions between the metals [33].

## Methods

### Study sample

Beginning in 2016, pregnant women and their newborns were recruited in delivery rooms of two hospitals in Israel: (1) Rambam Medical Center – the largest hospital in the Northern District of Israel, which accounts for around 5500 births annually, and (2) Shamir Medical Center – located in the Central region of Israel and which accounts for around 8000 deliveries annually. Women were considered eligible if they were Hebrew-speaking, aged 18 years or older, and pregnant with a singleton. Exclusion criteria included: (1) preterm birth (< 37 weeks of gestational age); (2) pregnancies considered by the medical staff to have a high risk of complications (e.g., autoimmune diseases, hypertension, diabetes) [34]; (3) minor or major congenital malformations as defined by the United States Centers for Disease Control and Prevention (CDC) and the European network of population-based registries for the epidemiological surveillance of congenital anomalies (EUROCAT) [35, 36]. A specialized study coordinator in each hospital obtained written informed consent from each woman before her participation and completed a questionnaire covering variables including sociodemographic characteristics, tobacco exposure, health status, pregnancy, and obstetric history. A total of 975 mother–newborns pairs were recruited from the hospitals: 509 from Rambam Medical Center and 466 from Shamir Medical Center. Maternal urine samples were collected from all participants on the day of delivery, and newborns’ anthropometric measures were taken by specialized neonatologists.

### Urinary metals and creatinine

Each participant was asked to provide a single urine sample. The samples were frozen at − 80 °C immediately after receiving them and then transported at − 20 °C for further analysis at the Central Public Health Laboratory of the Israeli Ministry of Health (Abu-Kabir). We measured levels of As, Cd, Cr, Hg, Ni, Pb, Se, and Tl using inductively coupled plasma mass spectrometry (ICP-MS), on an Agilent 7800 × ICP-MS instrument equipped with an Integrated Sample Introducing System (ISIS) and High Matrix Introducing mode (HMI). The procedure involved acid dilution of urine and direct injection into the ICP-MS instrument, followed by helium dilution in the HMI instrument. The method followed standard quality assurance and quality control procedures. Urinary metal concentrations were quantified using internal standard calibration procedures and certified analytical standards. Quality control was performed by analyzing aliquots of control material in each series (every ten samples), and accuracy was validated by the successful annual participation in the international proficiency test (G-EQUAS) for all parameters. Urine creatinine was measured using a well-established colorimetric method at the Central Teratology Laboratory at the Shamir Medical Center. It was used to standardize the metal concentration detected in the urine samples by a simple adjustment and a covariate in statistical models, as previously suggested by O'Brien et al. [37, 38].

### Newborns’ health and anthropometric measures

As part of a routine physical examination by trained neonatologists performed upon all infants following birth, birth weight, length, and head circumference were measured. The data were documented under an anonymized number for each mother–child pair. A total of 975 weight and head circumference measurements were conducted, as well as 887 length measurements. Each measurement was repeated three times for reliability, and mean values were computed. All results were documented in the newborns' medical records.

### Covariates

Using the comprehensive data collected from each mother via the questionnaires and data gathered from maternal medical registries, we were able to adjust our final models to account for possible confounders, including maternal age (continuous, in years), newborn’s gender, parity (nulliparous vs. multiparous), tobacco exposure during pregnancy (yes vs. no), socioeconomic status (SES) (standardized score), geographic area and creatinine concentration as measured in maternal urine. The maternal standardized SES index was individually calculated by matching maternally reported home address zip codes and the geographical distribution of SES as reported yearly by the Central Bureau of Statistics [39], using a geographical information system (GIS).

As gestational age could function as a mediator affecting the pathway between exposure and outcome [40], and therefore potentially lead to over- or under-estimation of the true effects [41], this variable was not included in the analysis.

Information on cigarette, cigar, or pipe smoking and the degree to which women were exposed to environmental tobacco smoke during pregnancy was self-reported by participants. Women were considered smoke-exposed if they reported either being an active smoker or were exposed to environmental tobacco smoke for 1 h or more per week during at least one-half of their pregnancy.

### Statistical analysis

Distributional plots and descriptive statistics were examined for all variables by the recruitment center (Rambam and Shamir). Mean values and standard deviations (SDs) were used to describe continuous variables, and independent t-tests were used to compare differences between groups. Median values, interquartile ranges (IQRs), and Mann–Whitney U tests helped describe and compare maternal urinary metal concentrations between groups. We used frequencies and chi-square tests to present and compare categorical variables between groups. All metal concentrations were modeled as natural log-transformed and standardized for IQR to achieve a standard scale and account for the positive skewness detected. The mean values of repeated anthropometric measurements were calculated and then standardized to the mean and SD of the study population.

For further analysis, statistical significance was two-sided and set at *p* < 0.05. All statistical processes were performed using R (version 4.1.1; R Foundation for Statistical Computing) and the data.table, ggplot2, dplyr, lubridate, and bkmr packages.

#### Multivariate linear regression

First, we evaluated the associations between exposure to individual metals during pregnancy and standardized anthropometric measures using multivariate linear regression models adjusted for maternal age, parity, newborn’s gender, tobacco exposure, SES, geographic area and creatinine concentration as measured in maternal urine. The standardized birth weight of newborns was also included as an independent variable in models that examined the association between exposure with birth length and head circumference. First, models were adjusted for covariates without considering interactions among the metals. Then, two- and three-way interactions of metal concentrations were included in the models. The results are presented as mean differences in SD of anthropometric measures (with 95% confidence intervals, CI) per IQR change in the log-transformed urine metal concentrations.

#### Bayesian kernel machine regression (BKMR)

Alongside the single pollution models, possible effects of joint exposures were examined. To examine potential interactions between metals on and their associations with the standardized birth weight, length, and head circumference, Bayesian kernel machine regression (BKMR) models were run. This novel non-parametric method enables a Bayesian variable selection framework to conduct analyses of mixtures without any prior assumption of linearity of the associations [33] and has been widely used in prenatal exposure studies [42–44]. Each model (Eq. 1) accounted for an anthropometric outcome, Y_i_, an independent exposure–response function, h(), as well as covariates (X_i_) and their corresponding coefficients (β).

1 $${Y}_{i}=h\left(A{S}_{i},C{d}_{i},C{r}_{i},H{g}_{i},N{i}_{i},P{b}_{i},S{e}_{i},T{l}_{i}\right)+ \beta {X}_{i}+{\epsilon }_{i}$$

In our study, BKMR models were fit using the Markov chain Monte Carlo algorithm, with 25,000 iterations using the Gaussian kernel [45]. All metals were entered into the model as one group, and the posterior inclusion probabilities (PIP) representing the contribution of each metal to the overall association were computed and reported.

For PIP, a minimal threshold of 0.50 was previously suggested [46] to determine whether a single exposure is important and has any substantial association with the estimates calculated via the models. Dose–response relationships were assessed jointly for all metals and individually for each metal by fixing other exposure agents at their median values. Further exposure–response relationships between the metals were explored as mean changes in the anthropometric measurements were calculated for IQR changes in the log concentration of each metal, while the concentrations of the other metals were fixed at their 25^th^, 50^th^, and 75^th^ percentiles. To further examine the possible bivariate metal–response associations, we visualized the anthropometric measures as functions of two exposures while concentrations of one metal change and the second were fixed at their 10^th^, 50^th^, and 90^th^ percentiles.

## Results

**Fig. 1:**
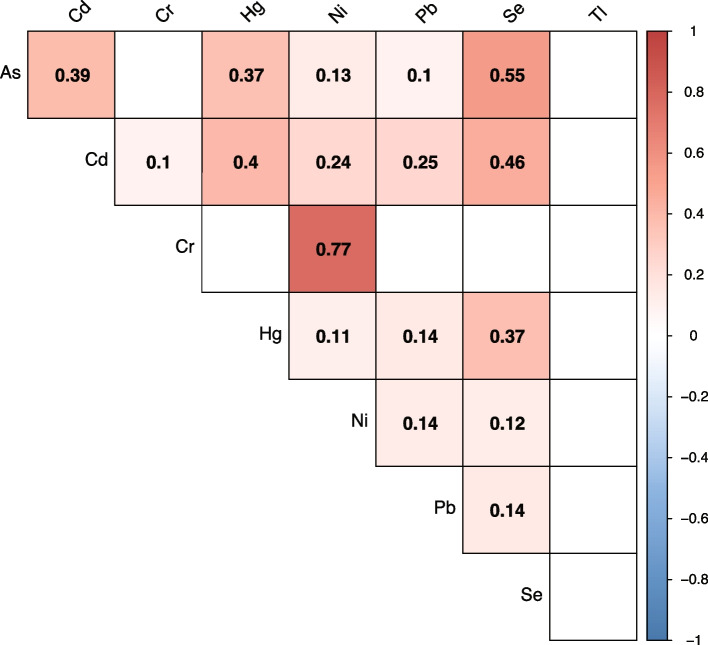
Pairwise Spearman's correlations matrix for metals concentrations (*n* = 975) from urine samples of study participants. Using a color spectrum, red indicates a positive correlation while blue indicants negative correlations. Only significant correlation (*p* < .05) coefficients appear in the figure. Metal concentrations were corrected for creatinine levels (μg/g), log-transformed, and were IQR standardized

Among 975 mother–newborn pairs recruited for the study (Table 1), the mean maternal age (SD) was 32.347 (4.580) years, and the mean (SD) gestational age at delivery was 39.472 (1.338) weeks; 509 newborns (52.2%) were male, and 466 (47.8%) were female. The mean birth weight (SD) was 3287.693 (441.475) g, the mean length at birth was 49.557 (2.203) cm, and the mean head circumference was 34.611 (1.272) cm. The overall metal concentrations, corrected for creatinine (μg/g creatinine), detected in maternal urine samples are shown in Table 2. Correlations between metals were tested, and Spearman’s coefficients are shown in Fig. 1.

**Table 1:** Participant sociodemographic, current pregnancy characteristics, and newborn’s anthropometric measures

Characteristic	Overall
**Maternal Age [years]**^1,3^	32.347 ± 4.580
**Parity**^1,4^	
Nulliparous	349 (36%)
Multiparous	626 (64%)
**Tobacco exposure**^1,4^	
No	937 (96.2%)
Yes	38 (3.9%)
**Socioeconomic Status Index**^1,3^	.527 ± .728
**Recruitment Center**^1,4^	
Rambam	509 (52.2%)
Shamir	466 (47.8%)
**Gestational age [week]**^1,3^	39.472 ± 1.338
**Newborn Gender**^1,4^	
Male	509 (52%)
Female	466 (48%)
**Newborn size for gestational age**^1,4^	
SGA	79 (8.1%)
AGA	833 (85.4%)
LGA	93 (6.5%)
**Newborn weight [gr]**^1,3^	3,287.693 ± 441.475
**Newborn length [cm]**^2,3^	49.557 ± 2.203
**Head circumference [cm]**^1,3^	34.611 ± 1.272

^1^*n* = 975

^2^*n* = 887

^3^Mean ± SD

^4^n (%)

**Table 2:** Distribution of metal concentrations corrected for creatinine levels (μg/g) as detected in maternal urine samples of participants (*n* = 975)

**Metal**	**LOQ**^1^	**% > LOQ**	$\frac{{\varvec{L}}{\varvec{O}}{\varvec{Q}}}{\surd 2}$	**AM**^2^	**GM**^3^	**25**^**th**^	**50**^**th**^	**75**^**th**^
As	0.02	99.79	0.01	18.31	9.61	4.82	8.64	17.35
Cd	0.02	66.26	0.01	0.17	0.11	0.05	0.14	0.24
Cr	0.02	89.54	0.01	0.64	0.27	0.17	0.28	0.49
Hg	0.01	85.23	0.01	0.33	0.16	0.08	0.17	0.38
Ni	0.02	91.18	0.01	2.68	1.42	1.15	1.89	3.05
Pb	0.04	69.03	0.03	0.46	0.23	0.12	0.25	0.49
Se	0.10	99.90	0.07	42.19	38.68	30.64	38.35	48.42
Tl	0.02	89.03	0.01	1.66	0.16	0.13	0.18	0.24

*Abbreviations*: *As* Arsenic, *Cd* Cadmium, *Cr* Chromium, *Hg* Mercury, *Ni* Nickel, *Pb* Lead, *Se* Selenium, *Tl* Thallium

^1^*LOQ* Limits of quantification [μg/L]

^2^*AM* Arithmetical Mean

^3^*GM* Geometrical Mean

### Multivariate linear regression analysis

**Fig. 2:**
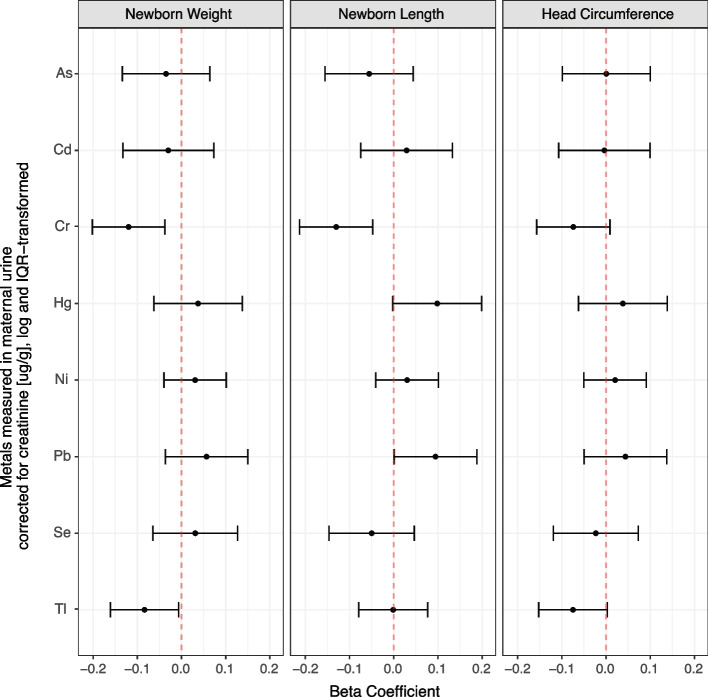
Z-standardized anthropometric measures as a function of single log-transformed IQR standardized metal concentrations (creatinine corrected). Linear models of weight (*n* = 975), length (*n* = 887), and head circumference (*n* = 975) are adjusted for parity, maternal age, tobacco exposure during pregnancy, standardized socioeconomic index, recruitment center, and creatinine levels

The linear regression results are shown in Fig. 2. When adjusting for covariates, a 1-IQR increase in log Cr concentration [μg/g creatinine] was associated with an average decrease of 0.120 SD (95% CI: -0.202 to -0.037; *P* = 0.004) in birth weight, as well as an average decrease in birth length of 0.133 SD (95% CI: -0.215 to -0.05; *P* = 0.002). A 1-IQR increase in log Tl concentration [μg/g creatinine] was also associated with an average decrease in birth weight, of 0.081 SD (95% CI: -0.158 to -0.004; *P* = 0.040). Head circumference was not significantly associated with any of the exposures. Neither two-way nor three-way significant interactions among the metals were detected for birth weight, length, and head circumference.

### BKMR analysis

**Fig. 3:**
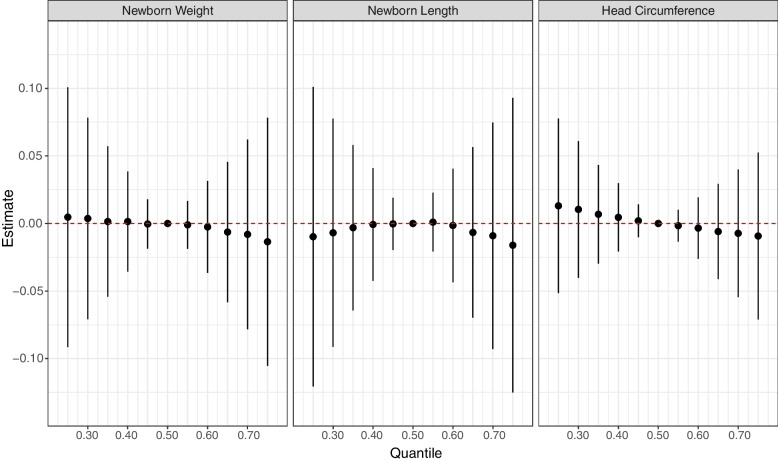
Joint effect (95% Credible Interval) of the mixtures on weight, length, and head circumference at birth by BKMR. All metals in a particular quantile were compared to all metals at their 50^th^ percentile. Models are adjusted for parity, maternal age, tobacco exposure during pregnancy, standardized socioeconomic index, recruitment center, and creatinine levels

**Fig. 4:**
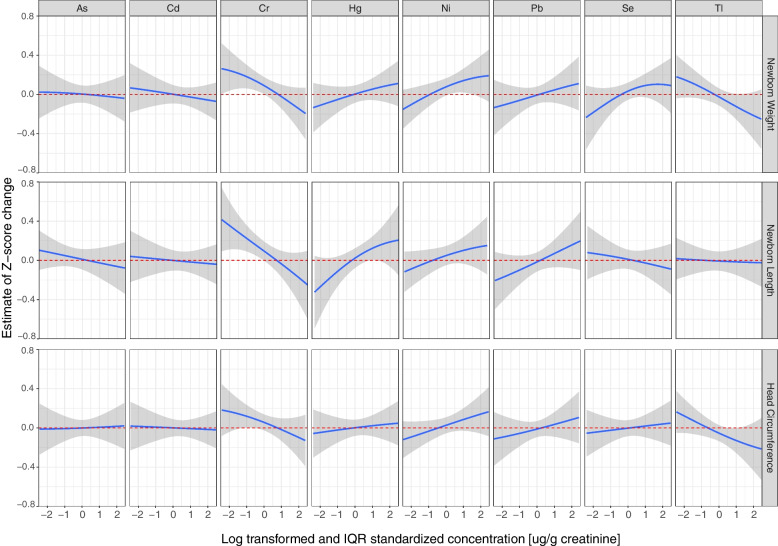
Univariate exposure–response models of anthropometric measures as function selected log-transformed IQR standardized metals (creatinine corrected). Weight (*n* = 975), length (*n* = 887), and head circumference (*n* = 975) are Z-standardized. The independently tested metal is scaled in each model, while the other metals are fixed at the 50th percentile. The gray area on the charts represents a 95% credible interval. Models are adjusted for parity, maternal age, tobacco exposure during pregnancy, standardized socioeconomic index, recruitment center, and creatinine levels

**Fig. 5:**
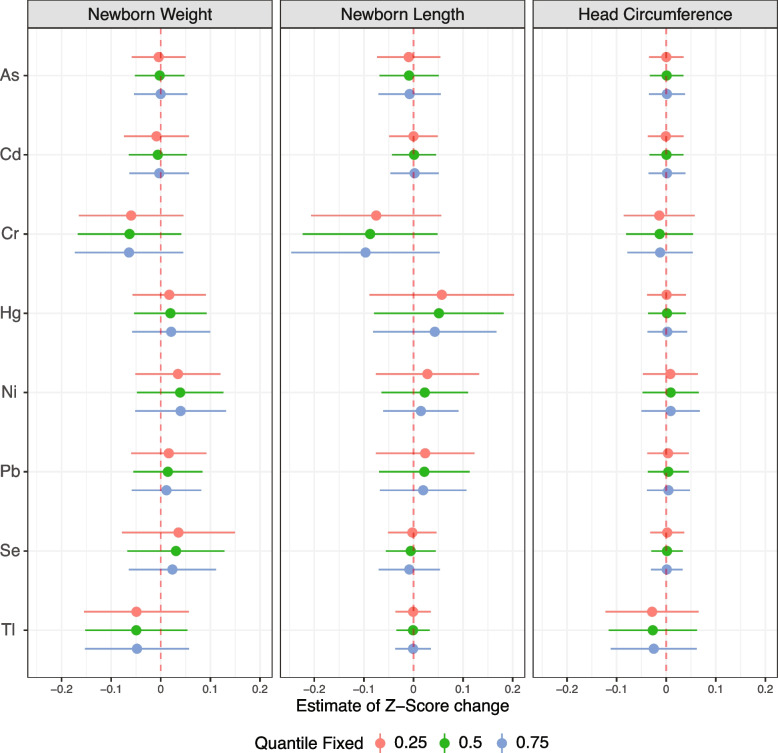
The individual exposure contributed to the overall effect of the metal mixture on z-standardized anthropometric measures. Weight (*n* = 975), length (*n* = 887), and head circumference (*n* = 975) are Z-standardized, while all metals (creatinine corrected) are log-transformed and IQR standardized. Using BKMR models, the individual contribution is indicated by the change in anthropometric estimates when exposure is at the 25th compared to the 75th percentile, while all the metals are fixed at either 25th, 50th, or 75th percentile (as indicated by Quantile fixed). The gray area on the charts represents a 95% credible interval. Models are adjusted for parity, maternal age, tobacco exposure during pregnancy, standardized socioeconomic index, recruitment center, and creatinine levels

BKMR was implemented to obtain estimates of the joint exposure–response function of all metals examined in our study. We first examined the overall mixture dose–response relationship with weight, length, and head circumference at birth (Fig. 3). The results suggest a negative association for weight and head circumference and a U-shaped association for length, yet all credible intervals obtained overlapped the null association. Then, the association of each creatinine-corrected metal (IQR-centered log concentrations) in the mixture was examined with weight, length, and head circumference at birth when all other metals were fixed at their median. Models were adjusted for the covariates mentioned above and are shown in Fig. 4. The PIP values of the birth weight model are shown in Table 3 and were above 0.5 for Cr, Tl, and Ni (0.757, 0.618, and 0.646, respectively); the other metals had PIPs between 0.30 and 0.49, suggesting the probability these metals had any substantial association with newborn weight was low, thus should be considered as if their influence on the predicted outcome was low. Similar to the findings obtained from the linear model of birth weight, an inverse association was found between Cr and Tl concentrations with birth weight, while Cr was the only metal that obtained a 95% credible interval that did not overlap zero. Positive linear associations were detected between Hg as well as Ni, Pb, and Se and birth weight, while As and Cd were negatively associated with birth weight. To further investigate possible effect modifications by metals, based on the non-linear associations detected, we estimated the associations of a 1-IQR increase in each metal while the other seven metals were fixed at their 25^th^, 50^th^, and 75^th^ percentiles (Fig. 5). A possible interaction was suggested if the estimates obtained for each metal varied while the concentrations of other metals remained unchanged. When examining the estimates of birth weight, no significant interaction among the metals was detected. For further investigation, we visualized two metals interactions plots in Figure S1 [see Additional File 1]. We denoted positive interactions as interactions in which higher levels of one exposure increased the slope of the association between the outcome and the other exposure. Hence, a positive interaction would attenuate a negative association with the outcome but would potentiate a positive association with the outcome, and vice versa for negative interactions. As shown in Figure S1 [see Additional File 1], there was a suggestion of a positive interaction between As and Cr that attenuated the negative associations between the metal and birth weight.

**Table 3:** BKMR Posterior Inclusion Probabilities (PIP) were obtained for each metal from models of anthropometric measures

Metal	Newborn Weight	Newborn Length	Newborn Head Circumference
As	0.303	0.174	0.135
Cd	0.350	0.131	0.105
Cr	0.757	0.772	0.198
Hg	0.467	0.524	0.139
Ni	0.646	0.313	0.159
Pb	0.434	0.254	0.136
Se	0.490	0.167	0.104
Tl	0.618	0.101	0.350

*Abbreviations*: *As* Arsenic, *Cd* Cadmium, *Cr* Chromium, *Hg* Mercury, *Ni* Nickel, *Pb* Lead, *Se* Selenium, *Tl* Thallium

Only two PIP values calculated for the metals in the length model were higher than 0.50 (Table 3): 0.772 for Cr and 0.524 for Hg. Length at birth appeared to be (Fig. 4) a decreasing function of Cr and an increasing function of Hg, yet only Cr function yielded a 95% credible interval that did not overlap zero. Further analysis (Fig. 5) did not show any susceptibility for interactions among the metals. An examination of the slopes obtained from the two-metal interaction plots (Figure S2 [see Additional File 1]) suggested a negative interaction between Cr and both Hg and Ni that potentiate the negative associations between the metals and attenuated the positive association between them and birth length.

Calculated PIPs for the head circumference model (Table 3) were lower than 0.5 for all metals. Although visualization of the univariate exposure–response chart (Fig. 4) did suggest a negative association of Cr and Tl with head circumference, but the credible intervals of both functions did overlap zero, suggesting a non-significant association. As shown in Fig. 5, the estimates obtained from the interaction models for each metal remained unchanged, suggesting no significant interactions among the metals. These findings were supported by the bivariate metal-response functions (Figure S3 [see Additional File 1]).

## Discussion

Using modeling approaches that account for linear as well as non-linear relations, we examined the association between eight metals detected in maternal urine at delivery and anthropometric measures of the newborns. Our findings suggested Cr had the most prominent negative association with weight and length. An inverse association between Tl and birth length was detected, while a positive association between Ni and birth weight was suggested.

The combined effect of all metals is shown and suggests a negative association for weight and head circumference and a U-shaped association for length. Although the trends can be visualized, the estimates are close to zero, and the credible intervals overlap the null association, suggesting the trends are insignificant. The joint effects, reflected by the crude trends may be influenced by biological and chemical interactions between the metals. Thus different associations can mask each other and should be further studied among larger sample sizes with higher exposure levels.

Our analysis suggested a negative association between increasing levels of Cr and a newborn's weight and length. Evidence of negative associations between Tl and Ni with birth weight and Hg with length was also detected. The reduction in birth weight associated with increased Cr levels was supported by both the linear and BKMR models, as the latter also suggested a positive interaction between Cr and both As and Se that attenuated the negative associations between the metal and birth weight. These interactions may explain the inconsistencies compared with other studies conducted in this field. Several studies have reported a possible decrease in newborn birth size and weight associated with increasing levels of Cr in maternal urine samples at birth [47] and during pregnancy [48]. However, other studies did not support these findings [49, 50], although none accounted for possible associations between the outcomes and mixtures of metals. There is increasing evidence to suggest that Cr in maternal blood is associated with placental insufficiency [51], increasing placental oxidative stress, and possible lower birth weight and pregnancy complications [52]. Besides this indirect mechanism, Saxena et al. [53] suggest that Cr can cross the placenta, accumulate in the fetal tissues, and could directly induce DNA damage [54] and affect intrauterine growth [48].

Although Se was non significantly associated with birthweight, the proximity of its calculated PIP to 0.5 and the possible interaction with Cr could not be neglected. Its possible association with the increase in birth weight is consistent with a study conducted by Solé-Navais et al. (2020) [55]. In their study, increased prenatal levels of Se detected in the blood of Norwegian pregnant women were found to be significantly and positively associated with birth weight. Monangi et al. (2021) [56] suggested that increasing levels of Se in maternal blood were associated with longer gestation and hence could contribute to the increase in birth weight. The mechanism underlying the involvement of Se in gestational duration is not fully understood. Still, it could be explained by its role in the suppression of mediators involved in the activation of labor in human fetal membranes and the myometrium [57]. The authors of another study [58], suggested Se could form chemical bonds, reduce the effect of teratogenic metals, and promote fetal growth. In our study, high concentrations of Se did appear to attenuate the reduction of birth weight associated with Cr. However, the mechanisms underlying this possible interaction and its association with anthropometric measures are beyond the scope of this study and should be further investigated.

Similar to Cr, increasing levels of Tl were significantly associated with lower birth weight, as shown in the linear models and supported by the BKMR models. These results are consistent with the findings of several studies [59–61], where Tl was found to be associated with decreased birth weight. It was previously suggested that Tl, as with Cr, can increase the placental as well as the fetal oxidative stress [62] and is thus associated with intrauterine growth restriction [63]. Prenatal exposure to Tl has been found to be associated with a decrease in maternal and fetal thyroid activity [64], which could be directly and indirectly related to developmental impairments [30]. However, while we found Tl levels were negatively and significantly associated with a newborn’s weight, they were not found to be associated with length or head circumference.

As shown in Table S2 [see Additional File 1], compared with other studies conducted in this field, the medians of most of the metal/creatinine concentrations (μg/g) detected in our study (Table 2) were lower [23, 60, 65–70], except for Se, which showed higher levels compared with other studies (geometric mean = 38.68 μg/g; median = 38.35 μg/g; IQR: 30.64–48.42 μg/g). However, this was similar to the amounts detected among pregnant women in the US [69] by Kim et al. (2019); geometric mean = 35.4 μg/g (IQR: 18.0–57.4 μg/g). The relatively low concentrations of metals detected in urine samples from our study population enabled us to examine possible associations between anthropometric measures at birth and prenatal exposure to metals at levels similar to the background averages.

Previous studies that examined the association of Ni with fetal growth have been inconclusive; however, several studies [71, 72] have reported positive associations between Ni and fetal development. The positive association between Ni concentration and fetal growth could be attributed to some of the nutritional benefits of Ni. As it has a biological function in metabolic pathways in which vitamin B12 is important [73], Ni could potentially affect the stages in fetal growth when consumption of B12 is enhanced [74]. The possible association between Ni, weight, and length was also observed by Howe et al. (2022) [71] and thus contributed the validity of our findings.

The association of maternal urine Hg concentrations with anthropometric measures of newborns has been investigated [58, 66]. While some studies did suggest an inverse association between prenatal exposure to Hg and anthropometric measurements at birth [75], most studies did not offer any significant association [58, 66] and were conducted among women exposed to median Hg levels five to six-fold higher than those observed in our study. In general, Hg levels detected in our study were lower than those seen among the US population [76] and significantly lower than the upper limit suggested [77] for pregnant women by the World Health Organization (WHO) (5–7 μg/g creatinine). In our study, Increasing levels of Hg were found to be positively but non-significantly associated with length. Yet, the Hg levels detected among participants in our study were low and had a narrow range (IQR = 0.08 to 0.38 μg/g creatinine) compared with other studies. Therefore, the associations with anthropometric measures should be considered carefully and studied further among populations with greater variances.

As Pb and Cd levels exceeded the limit of detection (LOD) in less than 70% of participants in our study and had a prominently lower range and mean compared with other studies [78–80], it is difficult to relate the dose–response relationships observed for these metals with changes in the anthropometric measures.

Previous literature on the association between As exposure and anthropometric measures of the newborn is relatively limited, and reports have had mixed findings: while some failed to reject the null hypothesis [81, 82], others reported an inverse association between increasing concentrations of As and birth weight [83], as well as birth size [84]. In our study, although non-significant, the association between As concentration in maternal urine and newborn weight was inverse and consistent with previous studies [30, 83]. As concentrations were not found to be associated with head circumference, similar to previous studies [85, 86]. The PIP value calculated for As suggested it did not influence the estimates calculated for the outcome. However, the findings in other studies were inconsistent; while Shih et al. (2020) [17] reported a positive association, other studies reported inverse [31, 87, 88] associations between As levels in maternal blood or urine and the head circumference of the newborn. Although we were failed to reject the null hypothesis, the inconsistency with previous studies highlights the need for further research.

None of the metals was found to be significantly associated with head circumference in any of the models run. Using the BKMR models for head circumference, the PIPs detected were less than 0.50 for all metals, and single exposure associations appeared insignificant. Various associations between metals and head circumference were previously shown by Rahman et al. (2021) [14], who examined associations between metals detected in maternal erythrocytes and newborn anthropometric measures. An infant’s head circumference was previously found to be associated with many prenatal and environmental factors, including the newborn’s gender and gestational age [89] and maternal nutrition [90]. However, it is predominantly determined by inheritance [90] and pathways involving many genes and transcription factors [91]; therefore alternations in head circumference characterize many genetic disorders [92, 93] and have been extensively studied. As many of the metals included in this study were previously found to act as genetic modifiers [94–96], suppressing or enhancing fetal expression of genes, it is not unreasonable to assume that interactions between these metals themselves [57], or with proteins [97], including transcription factors, could lead to various alterations in newborns’ phenotypes. Recent studies have suggested that metals could also interact with epigenetic processes that may be crucial to intrauterine development [98], especially in the context of metal mixtures. Investigating the biochemical mechanisms that contribute to genomic–metal interactions should be a key area for future research and might require the collection of samples such as placental tissue and cord blood.

The current study had several strengths: the large sample size, the examination of multiple metals, the use of classic as well as advanced mixture modeling analysis, and the heterogeneous population recruited from two different geographical areas and hospitals. However, there were also several limitations. Since maternal education and income data was not collected, individual zip code-based SES was used. As our study included only term newborns, any association between prenatal maternal exposure to metals and preterm deliveries could not be examined [56, 78, 87]. The metal concentrations observed in our study were relatively low; this enabled us to examine the possible effect of daily exposures. On the other hand, it limited the scope of outcomes associated with high concentrations and wide variances. Although metals could be measured in urine and were corrected to maternal hydration condition, they had a variety of half-lives, with some concentrations reflecting exposure that had occurred in the past few days (e.g., As, Ni, Pb, Se, and Tl), and others reflecting exposures over past weeks and months (e.g., Cd, Cr, and Hg) [99–102]. Thus, our findings cannot reflect any association between duration and prenatal timing of exposure with any of the anthropometric measures. It is worth mentioning that metals measured in urine did not reflect the existence of many possible potent forms in the human body, e.g., methyl-Hg [103], selenomethionine [104], and lead–protein complexes [105].

## Conclusion

Using a large sample size and multi-metal mixture data, we delineated a potential association between prenatal maternal exposure to heavy metals and newborns’ weight, length, and head circumference. Our findings suggested that Cr was the most influential metal in predicting weight and length, as it was also negatively associated with both. An inverse association between Tl and birth length was detected, while a positive association between Ni and birth weight was suggested. Although some findings were not consistent with those of other studies, the levels of heavy metals observed in our study were relatively low, with low variances. Hence some associations detected might be spurious and should be further investigated in future epidemiologic studies as well as in vitro and in vivo biochemical studies.

## Supplementary Information

**Additional file 1: Table S1.** Urinary Metal Concentrations (*n*=975) corrected for creatinine levels (μg/g) Stratified by recruitment center: Shamir and Rambam. **Figure S1.** Bivariate Exposure-Response Functions for z-standardized weight model (*n* = 975). **Figure S2.** Bivariate Exposure-Response Functions for z-standardized length model (*n* = 887). **Figure S3.** Bivariate Exposure-Response Functions for z-standardized head circumference model (*n* = 975). **Table S2.** BKMR Posterior Inclusion Probabilities (PIP) obtained for each metal from models of anthropometric measures including gestational age as an independent variable.

## Data Availability

The datasets generated and analyzed during the current study are not publicly available due to ethical restrictions but are available from the corresponding author upon reasonable request.

## Abbreviations

AGA: Appropriate for Gestational Age
As: Arsenic
BKMR: Bayesian Kernel Machine Regression
Cd: Cadmium
CDC: Center for Disease Control and Prevention
Cr: Chromium
EUROCAT: European network of population-based registries for the epidemiological surveillance of congenital anomalies
GIS: Geographic Information System
GM: Geometric Mean
Hg: Mercury
HMI: High Matrix Introducing Mode
ICP-MS: Inductively Coupled Plasma Mass Spectrometry
IQR: Interquartile Range
ISIS: Integrated Sample Introducing System
LGA: Large for Gestational Age
LOD: Limits of quantification
Ni: Nickel
Pb: Lead
PIP: Posterior Inclusion Probability
SD: Standard Deviation
Se: Selenium
SGA: Small for Gestational Age
SES: Standardized Sociodemographic Status
Tl: Thallium
