# Supplementary material for: Prenatal exposure to heavy metal mixtures and anthropometric birth outcomes: a cross-sectional study
Source: Environ Health. 2022 Dec 29;21:139. doi: 10.1186/s12940-022-00950-z (PMC9798586; doi:10.1186/s12940-022-00950-z)
Supplement: Supplementary file 1 — Additional file 1: Table S1. Urinary Metal Concentrations (n=975) corrected for creatinine levels (μg/g) Stratified by recruitment center: Shamir and Rambam. Figure S1. Bivariate Exposure-Response Functions for z-standardized weight model (n = 975). Figure S2. Bivariate Exposure-Response Functions for z-standardized length model (n = 887). Figure S3. Bivariate Exposure-Response Functions for z-standardized head circumference model (n = 975). Table S2. BKMR Posterior Inclusion Probabilities (PIP) obtained for each metal from models of anthropometric measures including gestational age as an independent variable. [file 12940_2022_950_MOESM1_ESM.docx]

**Supplemental Material**

**Table S1.** Urinary Metal Concentrations (n=975) corrected for creatinine levels (μg/g) Stratified by recruitment center: Shamir and Rambam.

| **Metals^1^ (μg/g)** | **Shamir**  **N = 466^2^** | **Rambam**  **N = 509^2^** | **p-value^3^** |
| --- | --- | --- | --- |
| As | 9 (5, 17) | 9 (5, 19) | .610 |
| Cd | .12 (.04, .21) | .16 (.06, .25) | <.001 |
| Cr | .23 (.13, .43) | .31 (.20, .53) | <.001 |
| Hg | .15 (.05, .38) | .20 (.10, .38) | <.001 |
| Ni | 1.59 (.81, 2.75) | 2.16 (1.48, 3.27) | <.001 |
| Pb | .30 (.16, .49) | .21 (.09, .48) | <.001 |
| Se | 38 (31, 49) | 39 (31, 48) | .960 |
| Tl | .19 (.14, .25) | .16 (.11, .23) | <.001 |

Abbreviations: As, Arsenic; Cd, Cadmium Cr, Chromium; Hg, Mercury; Ni, Nickel; Pb, Lead; Se, Selenium; Tl, Thallium.

^1^Metals corrected for creatinine levels

^2^Median (IQR)

^3^Mann-Whitney test

**Figure S1.** Bivariate Exposure-Response Functions for z-standardized weight model (n = 975). Each panel shows the association between the specified metal (columns) weight, setting a second metal (rows) to its 10th, 50th, and 90th percentile and all other elements to their median values, adjusting for parity, maternal age, tobacco exposure during pregnancy, standardized socioeconomic index, recruitment center and creatinine levels. Metals were log-transformed and IQR standardized. Abbreviations: As, Arsenic; Cd, Cadmium Cr, Chromium; Hg, Mercury; Ni, Nickel; Pb, Lead; Se, Selenium; Tl, Thallium.


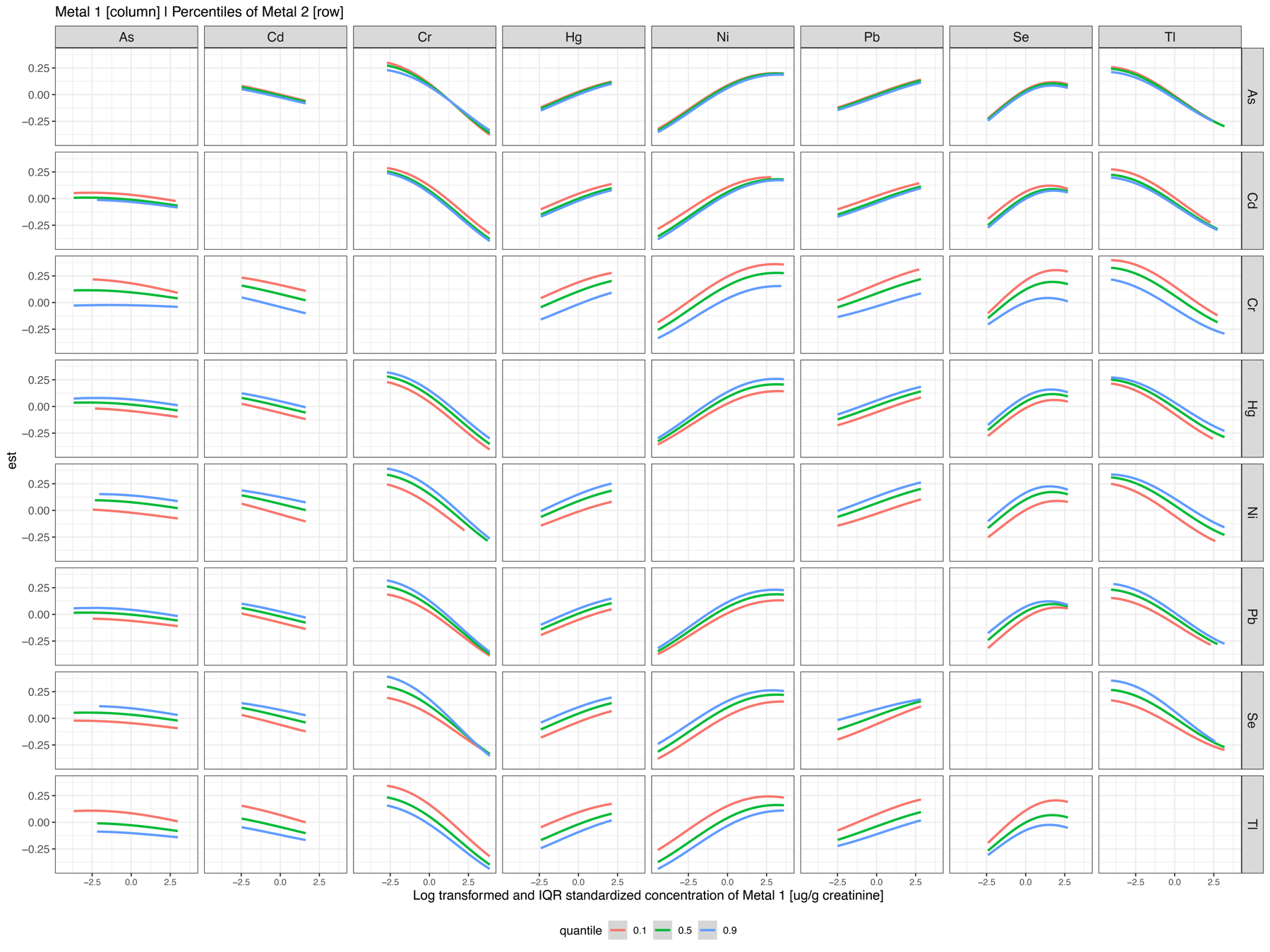


**Figure S2.** Bivariate Exposure-Response Functions for z-standardized length model (n = 887). Each panel shows the association between the specified metal (columns) length, setting a second metal (rows) to its 10th, 50th, and 90th percentile and all other elements to their median values, adjusting for parity, maternal age, tobacco exposure during pregnancy, standardized socioeconomic index, recruitment center and creatinine levels. Metals were log-transformed and IQR standardized. Abbreviations: As, Arsenic; Cd, Cadmium Cr, Chromium; Hg, Mercury; Ni, Nickel; Pb, Lead; Se, Selenium; Tl, Thallium.


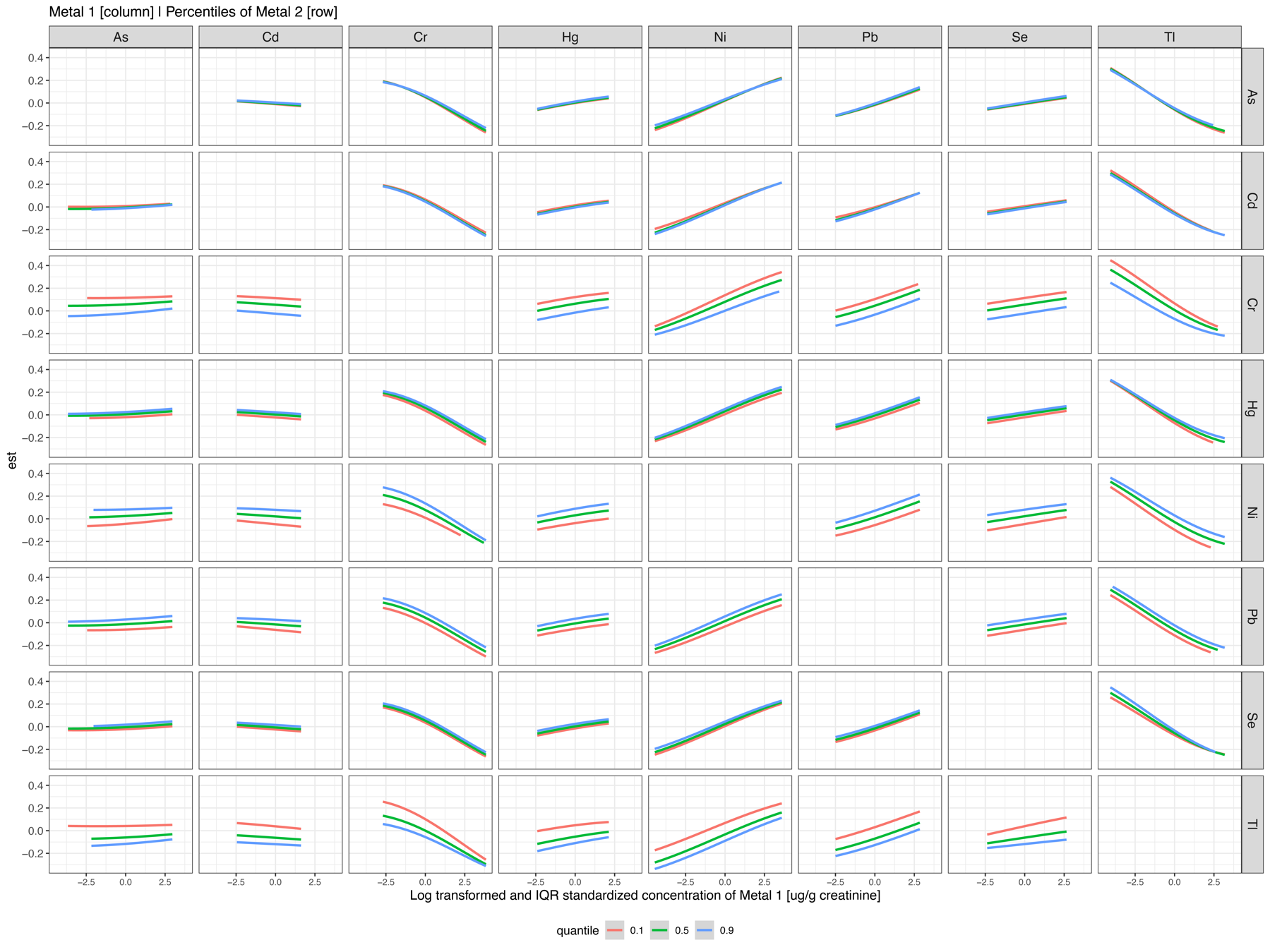


**Figure S3.** Bivariate Exposure-Response Functions for z-standardized head circumference model (n = 975). Each panel shows the association between the specified metal (columns) head circumference, setting a second metal (rows) to its 10th, 50th, and 90th percentile and all other elements to their median values, adjusting for parity, maternal age, tobacco exposure during pregnancy, standardized socioeconomic index, recruitment center and creatinine levels. Metals were log-transformed and IQR standardized. Abbreviations: As, Arsenic; Cd, Cadmium Cr, Chromium; Hg, Mercury; Ni, Nickel; Pb, Lead; Se, Selenium; Tl, Thallium.


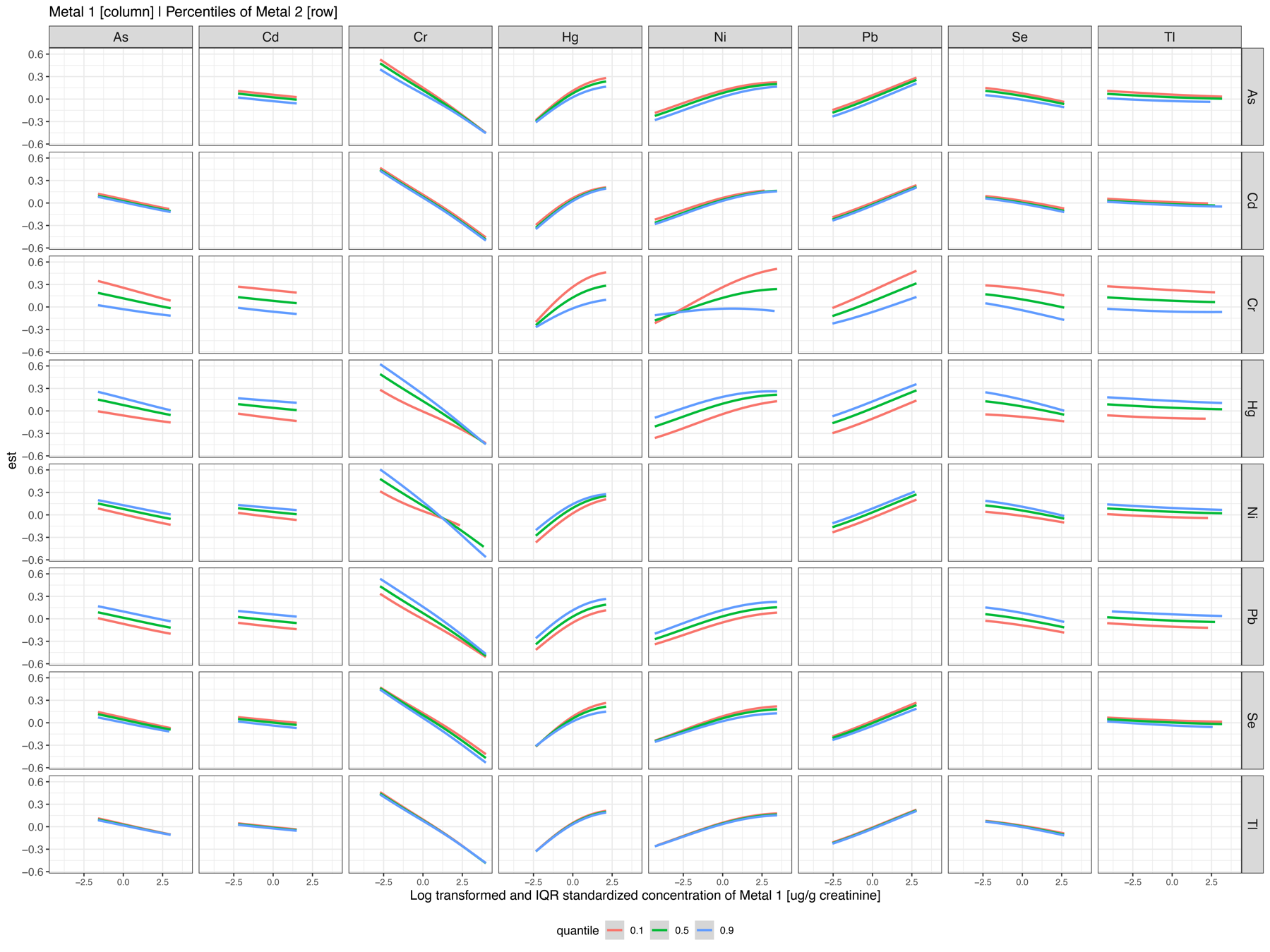


**Table S2.** BKMR Posterior Inclusion Probabilities (PIP) obtained for each metal from models of anthropometric measures including gestational age as an independent variable.

| **Metal** | **Reference** | **Location** | **Sampling year** | **N** | **Population** | **Arithmetic mean** | **median** | **Geometric mean** | **IQR^1^** |
| --- | --- | --- | --- | --- | --- | --- | --- | --- | --- |
| **As** | Wang et al. 2019^1^ | Wuhan, China | 2012–2014 | 7359 | Pregnant women | - | 28.22 | 30.49 | - |
|  | Kim et al. 2019^2^ | Boston, USA | 2006-2008 | 390 | Pregnant women | - | - | 15 | 8.19-31.7 |
|  | Wai et al. 2017^3^ | Ayeyarwady, Myanmar | 2015-2016 | 419 | Pregnant women | - | 74 | - | 45-127 |
|  | Fort et al. 2014^4^ | Spain | 2004-2006 | 489 | Pregnant women | 62 | 35 | - | - |
|  | Fang et al. 2021^5^ | Hubei, China | 2014-2015 | 831 | Pregnant women | - | 25.17 | - | - |
| **Cd** | Wang et al. 2019^1^ | Wuhan, China | 2012–2014 | 7359 | Pregnant women | - | 0.6 | 0.64 | - |
|  | Kim et al. 2019^2^ | Boston, USA | 2006-2008 | 390 | Pregnant women | - | - | 0.04 | 0.04-0.13 |
|  | Wai et al. 2017^3^ | Ayeyarwady, Myanmar | 2015-2016 | 419 | Pregnant women | - | 0.86 | - | 0.50-1.40 |
|  | Fort et al. 2014^4^ | Spain | 2004-2006 | 489 | Pregnant women | 0.67 | 0.54 | - | - |
|  | Fang et al. 2021^5^ | Hubei, China | 2014-2015 | 831 | Pregnant women | - | 0.8 | - | - |
| **Cr** | Kim et al. 2019^2^ | Boston, USA | 2006-2008 | 390 | Pregnant women | - | - | 0.2 | 0.09-0.28 |
|  | Fang et al. 2021^5^ | Hubei, China | 2014-2015 | 831 | Pregnant women | - | 1.25 | - | - |
| **Hg** | Bashore et al. 2014^6^ | New York, USA | 2007-2009 | 191 | Pregnant women | - | - | 0.32 | 0.24-3.50 |
|  | Barregard et al. 2021^7^ | Gothenburg, Sweden | 2021 | 31 | General population | - | - | 0.18 | - |
| **Ni** | Kim et al. 2019^2^ | Boston, USA | 2006-2008 | 390 | Pregnant women | - | - | 2.46 | 1.34-4.13 |
|  | Fort et al. 2014^4^ | Spain | 2004-2006 | 489 | Pregnant women | 4.8 | 3.9 | - | - |
|  | Fang et al. 2021^5^ | Hubei, China | 2014-2015 | 831 | Pregnant women | - | 4.27 | - | - |
| **Pb** | Wang et al. 2019^1^ | Wuhan, China | 2012–2014 | 7278 | Pregnant women | - | 3.44 | 3.69 | - |
|  | Kim et al. 2019^2^ | Boston, USA | 2006-2008 | 390 | Pregnant women | - | - | 0.3 | 0.10-0.58 |
|  | Wai et al. 2017^3^ | Ayeyarwady, Myanmar | 2015-2016 | 419 | Pregnant women | - | 1.8 | - | .0-3.3 |
|  | Fort et al. 2014^4^ | Spain | 2004-2006 | 489 | Pregnant women | 5.2 | 3.9 | - | - |
|  | Fang et al. 2021^5^ | Hubei, China | 2014-2015 | 831 | Pregnant women | - | 3.46 | - | - |
| **Se** | Wang et al. 2019^1^ | Wuhan, China | 2012–2014 | 7278 | Pregnant women | - | 17.61 | 18.24 | - |
|  | Kim et al. 2019^2^ | Boston, USA | 2006-2008 | 390 | Pregnant women | - | - | 35.4 | 18.0-57.4 |
|  | Wai et al. 2017^3^ | Ayeyarwady, Myanmar | 2015-2016 | 419 | Pregnant women | - | 23 | - | 18.0-30.0 |
|  | Fort et al. 2014^4^ | Spain | 2004-2006 | 489 | Pregnant women | 9.9 | 8.7 | - | - |
|  | Fang et al. 2021^5^ | Hubei, China | 2014-2015 | 831 | Pregnant women | - | 18.36 | - | - |
| **Tl** | Xia et al. 2015^8^ | Hubei, China | 2012–2014 | 816 | Pregnant women | 0.89 | 0.56 | - | - |
|  | Xiao et al. 2007^9^ | Guiyang, China | 2002 | 4 | General population | 0.65 | - | - | - |
|  | Wang et al. 2019^1^ | Wuhan, China | 2012–2014 | 7278 | Pregnant women | - | 0.53 | 0.55 | - |
|  | Kim et al. 2019^2^ | Boston, USA | 2006-2008 | 390 | Pregnant women | - | - | 0.1 | 0.05-0.18 |
|  | Fort et al. 2014^4^ | Spain | 2004-2006 | 489 | Pregnant women | 0.18 | - | - | - |
|  | Fang et al. 2021^5^ | Hubei, China | 2014-2015 | 831 | Pregnant women | - | 0.51 | - | - |

Abbreviations: As, Arsenic; Cd, Cadmium Cr, Chromium; Hg, Mercury; Ni, Nickel; Pb, Lead; Se, Selenium; Tl, Thallium.

^1^IQR = Interquartile range.

1. Wang X, Qi L, Peng Y, et al. Urinary concentrations of environmental metals and associating factors in pregnant women. Environ Sci Pollut Res. 2019;26(13):13464-13475. doi:10.1007/s11356-019-04731-z

2. Kim SS, Meeker JD, Keil AP, et al. Exposure to 17 trace metals in pregnancy and associations with urinary oxidative stress biomarkers. Environ Res. 2019;179:108854. doi:10.1016/j.envres.2019.108854

3. Wai K, Mar O, Kosaka S, Umemura M, Watanabe C. Prenatal Heavy Metal Exposure and Adverse Birth Outcomes in Myanmar: A Birth-Cohort Study. Int J Environ Res Public Health. 2017;14(11):1339. doi:10.3390/ijerph14111339

4. Fort M, Cosín-Tomás M, Grimalt JO, Querol X, Casas M, Sunyer J. Assessment of exposure to trace metals in a cohort of pregnant women from an urban center by urine analysis in the first and third trimesters of pregnancy. Environ Sci Pollut Res. 2014;21(15):9234-9241. doi:10.1007/s11356-014-2827-6

5. Fang X, Qu J, Huan S, et al. Associations of urine metals and metal mixtures during pregnancy with cord serum vitamin D Levels: A prospective cohort study with repeated measurements of maternal urinary metal concentrations. Environ Int. 2021;155:106660. doi:10.1016/j.envint.2021.106660

6. Bashore C, Geer L, He X, et al. Maternal Mercury Exposure, Season of Conception and Adverse Birth Outcomes in an Urban Immigrant Community in Brooklyn, New York, U.S.A. Int J Environ Res Public Health. 2014;11(8):8414-8442. doi:10.3390/ijerph110808414

7. Barregard L, Ellingsen DG, Berlinger B, Weinbruch S, Harari F, Sallsten G. Normal variability of 22 elements in 24-hour urine samples – Results from a biobank from healthy non-smoking adults. Int J Hyg Environ Health. 2021;233:113693. doi:10.1016/j.ijheh.2021.113693

8. Xia W, Du X, Zheng T, et al. A Case–Control Study of Prenatal Thallium Exposure and Low Birth Weight in China. Environ Health Perspect. 2016;124(1):164-169. doi:10.1289/ehp.1409202

9. Xiao T, Guha J, Liu CQ, et al. Potential health risk in areas of high natural concentrations of thallium and importance of urine screening. Appl Geochem. 2007;22(5):919-929. doi:10.1016/j.apgeochem.2007.02.008
